# Environmental, economic and socio-cultural risk factors of recurrent seasonal epidemics of cerebrospinal meningitis in Kebbi state, northwestern Nigeria: a qualitative approach

**DOI:** 10.1186/s12889-018-6196-9

**Published:** 2018-12-13

**Authors:** Semeeh A. Omoleke, Olatunji Alabi, Faisal Shuaib, Fiona Braka, Sisay G. Tegegne, Gregory C. Umeh, Johnson M. Ticha, Anthony Onimisin, Peter Nsubuga, Usman Adamu, Kabiru Mohammed, Chima Onoka, Wondimagegnehu Alemu

**Affiliations:** 1 World Health Organisation, Country Representative Office, Abuja, Nigeria; 2 WHO Kebbi State Field Office, Brinin Kebbi, Kebbi State Nigeria; 3 Department of Demography and Social Statistics, Federal University, Birnin Kebbi, Kebbi State Nigeria; 4 National Primary Health Care Development Agency, Abuja, Nigeria; 5 Global Public Health Solutions, Atlanta, GA USA

**Keywords:** Environmental, Socio-cultural and economic factors, Epidemic meningitis, Nigeria

## Abstract

**Background:**

Kebbi State remains the epicentre of the seasonal epidemic meningitis in northwestern Nigeria despite interventions. In this setting, no previous study has been conducted to understand the risk factors of the recurrent meningitis epidemics using qualitative approach. Consequently, this study intends to explore and better understand the environmental, economic and socio-cultural factors of recurrent seasonal epidemic meninigitis using a qualitative approach.

**Methods:**

We conducted in-depth interview (40 IDIs) and focus group discussions (6 FGDs) in two local government areas (LGAs) in Kebbi State, Northwestern Nigeria to understand the environmental, economic and socio-cultural factors of recurrent meningitis outbreaks. Routine surveillance data were used to guide the selection of settlements, wards and local government areas based on the frequency of re-occurrences and magnitude of the outbreaks.

**Results:**

The discussions revealed certain elements capable of potentiating the recurrence of seasonal meningitis epidemics. These are environmental issues, such as poorly-designed built environment, crowded sleeping and poorly ventilated rooms, dry and dusty weather condition. Other elements were economic challenges, such as poor household living conditions, neighbourhood deprivation, and socio-cultural elements, such as poor healthcare seeking behaviour, social mixing patterns, inadequate vaccination and vaccine hesitancy.

**Conclusion:**

As suggested by participants, there are potential environmental, socio-cultural and economic factors in the study area that might have been driving recurrent epidemics of cerebrospinal meningitis. In a bid to addressing this perennial challenge, governments at various levels supported by health development partners such as the World Health Organisation (WHO), United Nation Habitat, and United National Development Programme can use the findings of this study to design policies and programmes targeting these factors towards complementing other preventive and control strategies.

## Background

Nigeria is one of the countries that lie along the traditional African meningitis belt which spans from Senegal in the western part of the African continent to Ethiopia in the eastern part [1–3] Characteristically, cerebrospinal meningitis (CSM) epidemics start around February when the prevailing weather is hot and dry and tails off with the onset of rainfall in May or June [1–3].

Until recently, *Neisseria meningitidis type A* was the major cause of seasonal CSM epidemic within African meningitis belt [4–7]. However, other serotypes have been implicated in recent outbreaks in different parts of the belt, such as C, W135 and X [4, 6–8]. There are other bacterial pathogenic agents that are capable of causing an outbreak, however, on a smaller scale [4, 5].

Northern Nigeria has experienced recurrent seasonal CSM epidemics for several years, despite efforts being made by the government and development partners [5, 7, 9]. The recurrent CSM epidemics are of public health importance due to relatively high morbidity and case fatality rate, the huge population at risk and the over-stretching of the weak health services [7, 9, 10]. Most recently, between January and April, 2017, outbreak of type C meningitis was reported in northern states of Zamfara, Sokoto, Kebbi, Katsina, Niger and Yobe with it attendant high death toll.

Some interventions have been conducted in Northern Nigeria, including, a mass vaccination campaign against *Neisseria meningitidis type A*, reactive vaccinations against type C, episodic health education, and more recently, strengthening of laboratory surveillance for CSM [5, 7, 9]. Reactive vaccination is a vaccination response rapidly conducted to contain the spread of an epidemic within a defined geographic area.

Despite these efforts, the epidemic belt seems to have expanded in Nigeria from 26 to 29 states, and within states, for example, in Kebbi State, between 2014 and 2015, the number of affected Local Government Areas (LGAs) has increased from 10 to 16 [7]. Similarly, Kebbi State remains the epicentre of seasonal epidemics of meningitis in Northwestern Nigeria, given the magnitude and recurrent nature of the outbreaks [7].

Literature suggests some factors that underlie the spread of seasonal epidemic meningitis, though not fully elucidated [3]. For example, elements like climatic conditions- temperature, humidity, dustiness; previous history of meningitis; poor housing condition and household crowding; income and poverty level have been associated with seasonal epidemic meningitis. Most of these studies were not indigenous, and also, no recent published study has been done in Northern Nigeria to investigate and better understand the factors that may explain the recurrent nature of the seasonal scourge. Moreover, there is no study to the best of our knowledge that investigated these factors using a qualitative approach.

In the light of the above, we conducted a study to ascertain potential socio-cultural, economic and environmental factors that could enhance seasonal transmission of CSM in Kebbi State. The identified potential factors could be used to modify or reduce community vulnerabilities to CSM infection and transmission and could be complementary to the role of mass vaccination. Providing a more effective and multi-pronged intervention becomes imperative given the pathogenic shift observed post- mass vaccination campaign in 2013, a limited global stockpile of CSM vaccines for outbreak response and funding issues.

## Methods

### Study area

Kebbi state is one of the northwestern states of Nigeria with both local and international borders. Locally, it borders with Zamfara, Sokoto, and Niger States while it has international borders with Niger and Benin Republics. Kebbi State enjoys a tropical climate characterized by annual rainfall ranging from 800 mm (northern part of the state) and 1000 mm (in the south) while temperature ranges from 21 to 40 degree Celsius (mean temperature-26 degree Celsius). There are 21 Local Government Areas (LGAs) in the state with 225 political wards and four traditional Emirates. It has a projected population of 4,394,887 (2016) based on 2006 census population.

### Study population

**Fig. 1:**
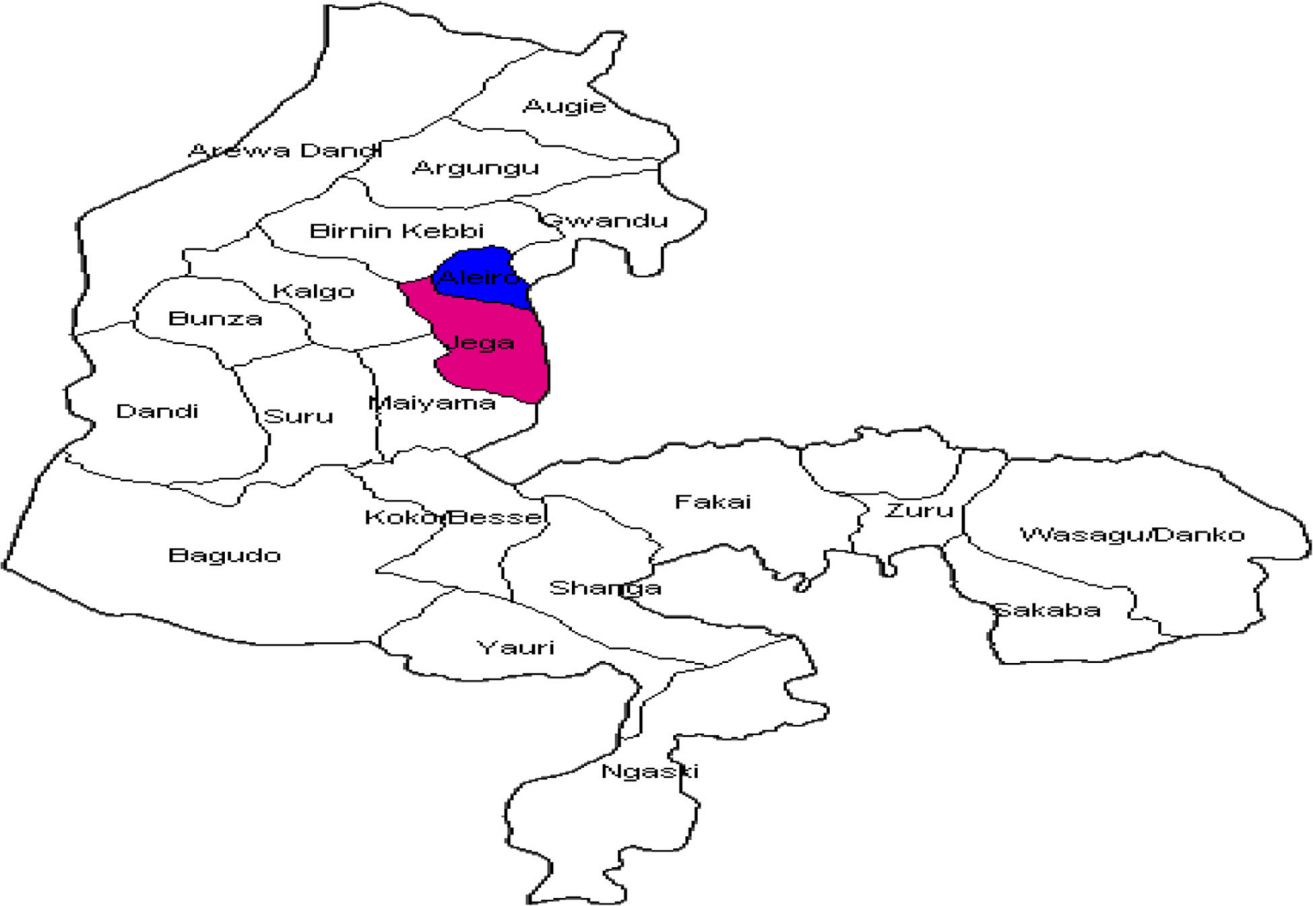
Map of Kebbi State showing the two Local Government Areas of interest (Aliero and Jega)

We selected Jega and Aliero LGAs of Kebbi State as investigation sites (please see Fig. 1 for the map of Kebbi State indicating the location of the LGAs of interest). Epidemiological data guided the selection- the LGAs have experienced repeated outbreaks of CSM for many years. These LGAs share many characteristics in common and interventions have been conducted in both LGAs aimed at addressing the repeated outbreaks. We identified eight settlements (five in Aliero and three in Jega LGA) from seven wards, where investigations were conducted as guided by epidemiological data. Interviews were conducted in 10 households per settlement.

### Study design

A qualitative research design was adopted using in-depth interviews and focus group discussion. We conducted 40 in-depth interviews (IDIs) and 6 Focus Group Discussions (FGDs) across the selected study areas to elicit information on the environmental, economic and socio-cultural factors driving meningitis outbreak in Kebbi State.

### Sampling technique

The study used purposive sampling techniques in selecting the LGAs, wards and participants. The LGAs with a history of recurrent meningitis outbreaks were purposively selected. Within the LGAs, most affected wards were further selected, and households within the selected wards were randomly selected and interviewed. Households were randomly selected in proportion to the size of the community as we were not able to do household listing.

### Data collection

We collected socio-demographic data of the respondents and selected household characteristics that have a bearing on predicting the likelihood of meningitis outbreak for each of the selected households. We also obtained information from health practitioners within the health facilities in the communities as well as the village heads.

We conducted in-depth interviews (IDIs) and Focus Group Discussions (FGDs) to elicit information on the environmental, economic and socio-cultural factors driving meningitis outbreak in Kebbi State. The choice of methods of data collection was informed by the type of data required. For instance, IDI was conducted with household heads or caregivers, Quranic School Mallams while FGDs were conducted with the village heads and health practitioners from the communities and health facilities in the communities. A total of 40 IDIs and 6 FGD sessions were conducted.

### Data analysis

We conducted data cleaning, processing and analysis manually using a thematic framework approach. This process involves verbatim transcription of returned audios by professional transcribers who were not part of the research team. The transcripts were screened, edited, and double-checked independently by the principal investigator. Transcripts were repeatedly checked for internal consistency, accuracy, and elimination of possible spurious responses using note-taking by the note taker during the interview sessions before further processing of the data. The moderator and note taker later screened the transcripts to ensure agreement on the content of the transcripts by the research team and the transcribers. Further, the data analysis involves coding, sorting, and theme identification. The coding log was developed based on the central objectives of the study, the research instrument, and various responses elicited from the participants.

We developed themes and sub-themes that are relevant to the greater understanding of the topic of the research. After coding, sorting and theme identification, we analysed the generated qualitative data using content analysis method. Content analysis involved a systematic compression of texts into replicable fewer content categories based on explicit rules of coding [11]. Thus, we summarized responses to each theme and sub-theme while important, and relevant expressions like quotations by the respondents were reported verbatim to enrich the output of the study.

## Results

### Participants’ characteristics

The age of participants ranged between 25 and 70 years. The predominant (60%) occupation among those employed is farming while the women were all housewives and were unemployed. There was a low level of education among participants in the LGAs studied; highest level of education was National Certificate in Education or Diploma level. Precisely 75% of the participants interviewed at the household level had Quranic education while 25% had primary school education (Table 1).

**Table 1:** Analysis of Key Variables from IDI using Frequency Table

S/N	Variables	Settlement: Jandutsi	Settlement: Birnin Yari	Settlement: Sarkin Fada II	Settlement: Attagarawa	Frequency
1	Number of people per room	1–3: 3	1–3: 4	1–3: 3	1–3: 4	14
		4–7: 4	4–7: 3	4–7: 4	4–7: 3	14
		8–10: 3	8–10: 3	8–10: 3	8–10: 3	12
2	Number of rooms available for sleeping	1–3: 6	1–3: 4	1–3: 6	1–3: 4	20
		4–7: 1	4–7: 3	4–7: 1	4–7: 2	7
		8–10: 3	8–10: 3	8–10: 3	8–10: 4	13
3	Housing arrangement	Space between building: 1	Space between building: 4	Space between building: 3	Space between building: 2	10
		Shared Toilet: 8	Shared Toilet: 5	Shared Toilet: 4	Shared Toilet: 4	21
		Single use: 1	Single use: 1	Single use: 3	Single use: 4	9
4	Size of the window in the room	Small: 6	Small: 6	Small: 4	Small: 10	26
		Medium:3	Medium: 3	Medium: 3	Medium: 0	12
		Big: 0	Big: 0	Big: 2	Big: 0	2
5	Size of the window in the kitchen	Small: 8	Small:8	Small: 10	Small: 10	36
		Big: 2	Big: 2	Big: 0	Big: 0	4
6	Location of kitchen	Close to the room: 5	Close to the room:5	Close to the room: 5	Close to the room: 5	20
		Far from the room: 4	Far from the room: 4	Far from the room: 4	Far from the room: 4	16
		Open Kitchen: 1	Open Kitchen:1	Open Kitchen: 1	Open Kitchen: 1	4
7	Type of cooking fuel used	Firewood: 10	Firewood: 5	Firewood: 10	Firewood/Millet Stalk: 10	35
			Gas:1			1
			Kerosene: 4			4
8	Neighbourhood wealth index	Middle class: 4	Middle class: 5	Middle Class: 7	Middle class: 3	18
		Poor: 6	Poor: 5	Poor: 3	Poor: 7	22
9	Educational status	Western: 0	Western: 4	Western: 0	Western: 6	10
		Qur’anic: 10	Qur’anic: 6	Qur’anic: 10	Qur’anic: 4	30
10	Social events	Market: 3	Market: 3	Market: 5	Market: 6	17
		Wedding: 2	Wedding: 4	Wedding: 2	Wedding: 0	8
		Naming ceremony: 2	Naming ceremony: 1	Naming ceremony: 0	Naming ceremony: 1	4
		Funeral: 0	Funeral: 0	Funeral: 1	Funeral: 0	1
		School: 2	School: 2	School:2	School: 0	6
		Farm: 1	Farm: 0	Farm:0	Farm: 0	1
		Majalisa: 0	Majalisa: 0	Majalisa: 0	Majalisa: 3	3
11	Often visits social gathering	Daily: 3	Daily: 4	Daily: 4	Daily: 5	16
		2-3 days/week: 5	2-3 days/week: 2	2-3 days/week: 3	2-3 days/week: 3	13
		4–7 days a week: 2	4–7 days a week: 4	4–7 days a week: 3	4–7 days a week: 2	11
12	Places for seeking health	Hospital: 1	Hospital: 6	Hospital: 3	Hospital: 10	20
		PMV: 4	PMV: 3	PMV: 5		12
		Traditional: 1	Traditional: 1	Traditional: 2		4
		Health worker (Unregulated Practice): 4	Unregulated Practice: 0	Unregulated Practice: 0	Unregulated Practice: 0	4
13	What influences treatment	Money: 7	Money: 9	Money: 5	Money: 5	26
		Severity of illness: 3	Severity of illness: 1	Severity of illness: 5	Severity of illness: 5	14
14	Who decides where to seek treatment	Father: 10	Father: 6	Father: 5	Father: 8	28
		Mother: 0	Mother: 4	Mother: 5	Mother: 2	11
15	How early after onset of symptoms	Immediately: 9	Immediately: 6	Immediately: 8	Immediately: 1	24
		Sometimes later: 1	Sometimes later: 4	Sometimes later: 2	Sometimes later: 9	16
16	Compliant to immunization	Yes:7	Yes: 8	Yes:7	Yes: 9	31
		No: 3	No: 2	No: 3	No: 1	9
17	Isolation of cases	Isolate infected person: 8	Isolate infected person: 8	Isolate infected person: 6	Isolate infected person: 8	30
		Avoid sleeping with people: 2	Avoid sleeping with people: 2	Avoid sleeping with people: 4	Avoid sleeping with people: 2	10
18	Environmental factors promoting for recurrent outbreak	Heat and dry weather: 7	Heat and dry weather: 7	Heat and dry weather: 7	Heat and dry weather: 10	31
		Sanitation: 3	Sanitation: 3	Sanitation: 3	Sanitation: 0	9
19	Factors that promote the spread of meningitis	Poor sanitation: 6	Poor sanitation: 6	Poor sanitation: 6	Poor sanitation: 2	20
		Poor ventilation: 4	Poor ventilation: 4	Poor ventilation: 4	Poor ventilation:8	20

### Theme: Environmental factor as a driver or risk factor for CSM transmission

#### Sub-theme: Crowded sleeping space, built environment, and weather conditions

The results showed that 26 respondents, constituting 65%, mentioned that four to ten persons sleep in one room in multi-dwelling apartments with poorly ventilated rooms. There were 31 (78%) respondents during IDI who indicated that heat/or dry and dusty weather condition appears to be a trigger for the epidemic (Table 1). The finding from one of the FGDs was quite instructive regarding the impact of poor ventilation on health:

*“Some build rooms without windows thinking it is more secure but indirectly killing themselves………..”* –FGD from Aliero LGA

The participants during FGD linked the onset and end of the epidemic to prevailing weather condition:

*“The disease usually starts when the weather is very hot and dry, and ends when rains start, usually in June or around this time”* - FGD from Jega LGA

Further, the presence of poor sanitation, congestion of structures and building design- kitchen or cooking space being close to living rooms as part of the environmental problems confronting the communities were captured during IDI (Table 1).

In an attempt to provide information about the seasonality and recurrent nature of the outbreak, the participants (health workers) unanimously agreed that the 2014 outbreak was massive but declined in 2015.

*“When the rains drop fully, CSM outbreak disappeared in 2014 and 2015, but the worst record of outbreak in recent years was in 2014….”-* FGD from Aliero LGA

### Theme: Economic factors

Table 1 showed that 22 (55%) respondents interviewed during IDI asserted that poor household income and poverty in the neighbourhood are rife. It affects their decisions regarding choice of care and timing of seeking care.

Also, analyses of the socio-economic indicators, such as level of education, employment status, neighbourhood wealth index and cooking fuel type were captured. None of the participants interviewed had university degree while 30 (75%) respondents interviewed during IDI had Quranic education, followed by primary school education. All the women participants were housewives who are un-employed. Other participants were mainly farmers (often non-mechanised). Also, 35 (88%) of them used firewood to cook, and to some extent, millet stalks.

### Theme: Socio-cultural factors

#### Sub-theme: Healthcare seeking practices- who decides, when and where do they seek care?

Table 1 showed that 35 (88%) respondents stated that father (household head) decides when and where they seek medical care. Similarly, 24 (55%) respondents sought orthodox care when the disease becomes severe while alternative care sources, such as patent medicine vendor/chemist, unlicensed health workers and traditional healers are the first choice. This finding was reinforced by results from FGD as shown below (see also summary in Table 2):

*“Most of the households seek treatment for many ailments through alternative health care providers (traditional and spiritual healers) and patent medicine vendors or graduates of the school of health technology who are not employed. We do not have a long waiting period and we spend less money compared to a government hospital. Some ‘local doctors’ also come home to provide treatment. It is when this fails that we patronize the general hospital. Parental care is also poor in the community- children are mostly not given proper attention and mothers are not in a position to take decision about where to treat and many of them are not employed”-* FGD (Traditional Leaders)

**Table 2:** Summary of some of the key results from interviews and FGDs conducted at Aliero and Jega LGAs

Themes	Sub-themes	Responses
Environmental factor as a driver or risk factor for CSM transmission	a) Crowded sleeping space	*“Most of our households have large family size and about 4–7 persons sleep in a room, making the living environment dirty”-*IDI from Aliero LGA
	b) Timing of onset of epidemics	*“The disease usually starts when the weather is very hot and dry, and ends when rains start, usually in June or around this time”* - FGD from Jega LGA
	(c) Built environment	*“Poor ventilation, congestion of structures such as kitchen and toilet facing each other or close to each other, some do not even use the kitchen but cook their food very close to sleeping rooms making it very hot. Lack of community hygiene is also important”-*FGD Aliero LGA
Economic factors	(a) Household income	*“Poor environmental sanitation, lack of vaccination to prevent the menace, poor government law to control waste, hunger (malnutrition) and poverty are some of the factors that encourage the seasonal occurrence of CSM”-* FGD from Jega LGA
	(b).Neighbourhood	“Many of the households here are poor and the neighbourhood receives little government attention”- FGD
Socio-cultural factors	(a).Health seeking practices	*“Most of the households seek treatment for many ailments through alternative health care providers (traditional and spiritual healers) and patent medicine vendors or graduate of the school of health technology who are not employed. We do not have a long waiting period, and we spend less money compared to a government hospital. Some ‘local doctors’ also come home to provide treatment. It is when this fails that we patronize the general hospital. Parental care is also poor in the community- children are mostly not given proper attention and mothers are not in a position to take decision about where to treat and many of them are not employed”-* FGD (Traditional Leaders)
	(b). Social mixing patterns	*“As you know, women in our environment usually marry early, so I attend wedding ceremony most weekends, and this also provides the opportunity to meet family and friends from villages and other towns”-* IDI *“I am an onion farmer, and we receive buyers from within Nigeria and the neighbouring Niger Republic, it is a big market, and we mix freely with them even during the outbreak period”-* IDI
	(c). Vaccination	*“We usually refuse vaccination here in this place but this is changing because we now understand the importance of vaccination, especially to children. Though, truly, there are still parents who do not believe in vaccination”-* FGD from Jega LGA *“There is a poor attitude to medical check-up and no routine vaccination against cerebrospinal meningitis…”-* FGD from Aliero LGA

#### Sub-theme: Social mixing patterns

Table 1 showed the frequency of visits and the major activities where participants mingle with other people, usually in a crowded environment, include markets, wedding ceremony, burial, majalisa (local parliament) and women islamiyyah. Further insight into the pattern and motivation behind the social arrangements were expressed below (see also summary in Table 2):

*“As you know, women in our environment usually marry early, so I attend wedding ceremony most weekends, and this also provides the opportunity to meet family and friends from villages and other towns”-* IDI

One of the farmers also emphasized that *“I am an onion farmer and we receive buyers from within Nigeria and the neighbouring Niger Republic, it is a big market, and we mix freely with them even during the outbreak period”-* IDI

Table 1 also showed that 30 (75%) IDI respondents mentioned that infected person with CSM are not usually isolated from healthy individuals when being nursed at home. The finding from FGD corroborated the IDI results as stated below:

*“Overcrowding of children in a room, lack of windows that lead to poor ventilation, lack of hygiene, mixing with infected persons are common findings that enhance the spread of the CSM in this environment”*- FGD Aliero LGA

#### Sub- themes: Attitude to vaccination

Some of the respondents take vaccination and are aware that vaccination is one of the ways of preventing disease transmission while some are still hesitant. A clue to the communities perception of vaccination was captured in the IDI as quantified in Table 1. This finding was reinforced during the FGDs (see summary in Table 2):

*“We usually refused vaccination here in this place, but this is changing because we now understand the importance of vaccination, especially to children. Though, truly, there are still parents who do not believe in vaccination”-* FGD from Jega LGA

Insight into scarcity of routine vaccination against CSM was mentioned as part of the problem in preventing and controlling the epidemic in one of the FGDs. The participants expressed their opinions as thus:

*“Poor environmental sanitation, lack of vaccination to prevent the menace, poor government law to control waste, hunger (malnutrition) and poverty are some of the factors that encourage the seasonal occurrence of CSM”-* FGD from Jega LGA

## Discussion

Our study revealed the presence of favourable environmental, socio-cultural and economic factors linked to the spread of recurrent meningitis in Kebbi State.

Our findings showed that the affected communities were aware and appreciated the existence of a temporal relationship between onset of the seasonal epidemic and the prevailing hot and dusty weather while the falling of the rains signified the end of the epidemic. A similar finding was reported in 2014 by Codjoe and colleagues, who conducted an FGD to elicit the perception of communities about the relationship between climatic conditions and the CSM epidemics [12] . They reported that “the majority of participants rightly linked CSM infections to dry, very hot and dusty conditions experienced during the dry season” [12]. Other studies have used different techniques to provide the relationship between CSM infection and certain favourable climatic conditions, such as dusty and windy atmosphere, long period of dry and hot weather, low rainfall and low humidity [13–15] .

The literature suggests that deforestation activities expose affected areas to the ventilation of aerosols and dust, thus increasing the risk of transmitting meningococcal infection [13, 16]. The impact of poor urban planning and building control reflects as the houses in the studied communities had poor ventilation (mainly small window size, person per room varies from 4 to 10) and congested structures. Our finding is in tandem with evidence from existing literature [12, 17] implicating the poorly ventilated rooms and congestion. However, Feraro and colleagues did not find an association between household crowding and self-reported respiratory symptoms in a study conducted in seven countries within the African meningitis belt [18]. This disparity in findings might be explained by the “unrestrictive definition” (those who eat from the same pot) of household used in the study [18].

The information from participants clearly demonstrated the role of poor economic condition as a risk factor for recurrent epidemic meningitis in the study areas (Aliero and Jega LGAs). The role of economic factors has been documented in the literature regarding resilience or individual and community vulnerabilities to infectious disease transmission [19–21]. Specifically, some studies have linked poor household income, unemployment, use of firewood for cooking and neighbourhood social deprivation role in the perpetuation of respiratory diseases and other communicable diseases epidemic [19–21]. There exists widespread poverty at household levels, and many of the neighbourhoods are socially-deprived with poor sanitation, poor nutrition, low level of education and massive unemployment as well as a disproportionately high number of low-income earners. We also observed that the major source of cooking fuel was firewood and could have a role in the spread of airborne infections or respiratory tract infections such as CSM. Some of the component of the fumes may stimulate inflammation, coupled with the dry and hot weather, and then facilitate the invasion of pathogenic meningococcal meningitis. All the economic indices at household and neighbourhood levels were poor. Hence, the communities are quite vulnerable to diseases, including recurrent meningitis.

Based on the results of this study, some socio-cultural factors seemed to be responsible for the recurrent transmission of epidemic *Neisseria meningitidis* in the study locations. For example, we identified that most women are housewives, usually with little or no formal education and unemployed, hence their inability to make informed decisions on family health. For instance, the decision to accept vaccination or when and where to seek medi-care rest essentially on the husband (men).

There seemed to be a preponderance of opinions regarding the use of alternative healthcare providers as a first referral centre. This choice was influenced by cultural practices and poor socio-economic status. Furthermore, patronage of government hospital was considered as the last resort- when the illness became severe and not amenable to alternative care.

Further, the local socio-cultural environment supports multi-dwelling housing, for instance, it is permissible for young couples to be accommodated at the family house (where the parents reside). The practice breeds over-crowding and over-stretches the available housing facilities. Multi-dwelling living pattern among close family members was widely reported in the course of our investigation in the communities surveyed. The socio-cultural practice has implications on family resilience to air-borne diseases like CSM, and general health and well-being of family or community.

The role of social mixing patterns was ascertained as a risk factor in this paper. For example, we found that even in the midst of outbreak, onion market which serves both local and international traders still opens without any form of precautionary measures.

There was a modest level of social interaction in the study areas. Most of the participants frequently engaged in social activities, such as wedding, market, burial, Islamic learning centre. These spaces were often crowded with people and often served as channels for infectious disease transmission. Previous studies or reports have also linked social mixing pattern to the spread of respiratory tract infections, including meningitis [17, 22].

Substantial progress has been made by WHO and other partners to create demand for routine and supplementary immunisation in Kebbi State [23]. Jega LGA (one of the two LGAs studied) was particularly known for non-compliance to immunisation as it was selected among the 77 LGAs that benefitted from various demand creation interventions aimed at making polio vaccines attractive and simultaneously, addressing other needs of the communities [23]. The existence of chronic non-compliance is not limited to polio immunisation (perhaps, worse with Oral Polio Vaccine) but also applicable to other routine or supplementary antigens. The social misconception came to fore during the 2015 reactive vaccination for meningitis where multiple demand creation interventions had to be instituted, even in the midst of the outbreak, to increase vaccination coverage. Non-compliance or vaccine hesitancy is an important social and communication barriers, and this has made control efforts difficult in the recent past.

We recognized the following limitations, though these might not have adversely affected the outcome of the study. Many of the participants did not understand English. Hence, translations were undertaken and might have introduced bias into the study findings. We were limited by the depth of knowledge of the participants in some aspects of the interview that are plausibly related to the recurrent transmission, for example, the role of deforestation and climate change. Further, we observed that the knowledge of the research assistants conducting the interviews seemed relatively limited and might have affected the quality of probing, and in turn, the depth of response from participants. Lastly, we did not fully explore the role of some social behaviours (e.g., smoking, going to clubs and bars) in the spread of CSM in this paper, though some of these elements are rare in the communities investigated.

Despite the limitations, we concluded that there were favourable environmental, socio-cultural and economic factors in the study areas that might have been driving recurrent epidemics of CSM. In a bid to addressing these recurrent CSM epidemics, the governments at various levels supported by health development partners can use the findings of this study to design policies and programmes targeting these factors towards complementing other preventive and control strategies.

## Conclusion

As suggested by participants, there are potential environmental, socio-cultural and economic factors in the study area that might have been driving recurrent epidemics of cerebrospinal meningitis. In a bid to addressing this perennial challenge (recurrent seasonal CSM epidemic), governments at various levels supported by health development partners such as the World Health Organisation (WHO), United Nation Habitat and United National Development Programme can use the findings of this study to design policies and programmes targeting these factors towards complementing other preventive and control strategies.

## Abbreviations

CSM: Cerebrospinal Meningitis
FGD: Focus Group Discussion
IDI: In-depth Interview
LGA: Local Government Area
WHO: World Health Organisation
